# Kawasaki disease patients homozygous for the rs12252-C variant of interferon-induced transmembrane protein-3 are significantly more likely to develop coronary artery lesions

**DOI:** 10.1002/mgg3.79

**Published:** 2014-05-14

**Authors:** Neil E Bowles, Cammon B Arrington, Keiichi Hirono, Tsuneyuki Nakamura, Long Ngo, Yin Shen Wee, Fukiko Ichida, John H Weis

**Affiliations:** 1 Division of Cardiology, Department of Pediatrics, University of Utah School of MedicineSalt Lake City, Utah; 2 Department of Pediatrics, Faculty of Medicine, University of ToyamaToyama, Japan; 3 Department of Pediatrics, Kanazawa Medical UniversityKanazawa, Japan; 4 Department of Medicine, Harvard Medical School, Beth Israel Deaconess Medical CenterBrookline, Massachusetts; 5 Department of Pathology, University of Utah School of MedicineSalt Lake City, Utah

Kawasaki disease (KD) is the most common systemic vasculitis syndrome, primarily affecting small- to medium-sized arteries, more particularly the coronary arteries (Kato et al. 1996). KD was first described in 1967 and is now identified as the leading cause of acquired heart disease among children in developed countries (Wang et al. 2005). The annual incidence of KD in children of Japanese descent is about 218 per 100,000 children less than 5 years of age (Nakamura et al. 2012) as compared to about 20 per 100,000 in the United States (Holman et al. 2010a). Timely treatment with high-dose intravenous γ globulin (IVIG) reduces the duration of fever and incidence of coronary artery lesions (CAL). However, even after IVIG treatment ∼5–7% of patients develop aneurysms (Ogata et al. 2013).

It is widely believed that KD is induced by one or more infectious agents that evoke an abnormal immunological response in genetically susceptible individuals (Burgner and Harnden 2005). However, since the initial description of KD, identification of a definitive infectious agent has been elusive. Several lines of evidence support the infection hypothesis including the acute onset of a self-limited illness, increased susceptibility at younger age, and geographic clustering of outbreaks with a seasonal predominance (later winter and early spring) (Wang et al. 2005).

There is a higher incidence of KD in Japan as well as among Japanese descendants residing in the United States than in any other ethnic populations (Holman et al. 2010b), suggesting that a genetic predisposition also plays an important role in susceptibility to the disease. In addition, there is evidence that the incidence of KD in parents and siblings of an affected patient is higher than in the general population (Onouchi 2012). For example, it has been reported that siblings of affected children are at 10- to 30-fold greater risk of developing KD than children in the general population (Fujita et al. 1989). In addition, offspring of individuals diagnosed with KD are more likely to develop KD (Uehara et al. 2004). More recently there have been a large number of genetic linkage and genome-wide association studies (GWAS) that have reported genetic loci associated with risk and outcomes, see Onouchi (2012) for a comprehensive review. Among the loci that have been implicated in large GWAS studies and have been replicated by separate studies are *FCGR2A* (Khor et al. 2011; Onouchi et al. 2012), *CASP3* (Onouchi et al. 2010; Kuo et al. 2013), and *BLK* (Onouchi et al. 2012; Chang et al. 2013).

HLA-B haplotypes have also been linked to KD with one study identifying KD-associated polymorphisms in *ABHD16A* (abhydrolase domain containing 16A; also known as BAT5: HLA-B associated transcript 5) (Hsieh et al. 2010), this association has not been replicated by other studies. *ABHD16A* encodes a highly conserved, widely expressed lipase of unknown specificity although it has been proposed to function as a palmitoylthioesterase (Martin et al. 2012). ABHD16A binds to IFITM1 (interferon-induced transmembrane protein 1) (Lehner et al. 2004). Another member of the family, IFITM3 (OMIM: 605579), is transcriptionally induced by type I and II interferons and serves to block cellular infection by viruses (such as influenza and dengue) that require endosomal entry into the cytoplasm for replication (Brass et al. 2009; Jiang et al. 2010; Weidner et al. 2010; Lu et al. 2011).

An allelic variant in the human *IFITM3* gene (SNP rs12252: NM_021034.2:c.42T>C; p.Ser14=) truncates the first 20 amino acids of the protein by introducing an alternative splice site and results in the loss of its “anti-viral” function (Everitt et al. 2012). Everitt and colleagues also showed that for European Caucasian patients infected with influenza A H1N1/09 virus, those homozygous for the C allele were significantly more likely to develop severe infections requiring hospitalization. More recently, Zhang et al. (2013) made a similar observation in Chinese patients infected with H1N1/09 influenza. The objectives of the study were (1) to evaluate for differences in *IFITM3* genotype frequencies between KD and control cohorts, (2) to assess whether there are differences in the incidences of CAL among the three KD genotypes, and (3) to assess for differences in the distributions of demographic factors (age, gender), IVIG treatment, laboratory data (C-reactive protein [CRP] levels and numbers of white blood cells [WBC]), and duration of fever.

In this study, we genotyped 140 KD patients recruited at three centers, the University of Toyama (*n* = 89), Kanazawa Medical University (*n* = 10), and the University of Utah (*n* = 41), for the rs12252 SNP. Patients were diagnosed with KD according to standard diagnostic criteria (Kawasaki et al. 1974; Kawasaki 1979). All patients were treated with IVIG and oral aspirin at the time of diagnosis. Echocardiography was used to determine whether the patients had developed CAL, defined as a coronary artery with a diameter of 3 mm or more (4 mm if the subject was over the age of 5 year) at ≥1 month after the onset of KD (Shulman et al. 1995).

With informed consent, venous blood samples or buccal swabs were obtained at the time of diagnosis and DNA isolated and stored at −20°C. For genotyping, both coding exons of *IFITM3* were amplified from 10 ng of genomic DNA using Platinum *Taq* polymerase (Life Technologies, Carlsbad, CA) (Arrington et al. 2012) and the oligonucleotide primers, IFITM3_1_F: 5′-CAAATGCCAGGAAAAGGAAA-3′ and IFITM3_2_R: 5′-CGAGGAATGGAAGTTGGAGT-3′. The 1158 bp PCR product was analyzed by agarose gel electrophoresis, purified by treating with Exo-SAP-IT (Affymetrix, Santa Clara, CA), and then submitted to the University of Utah DNA sequencing core for analysis (Arrington et al. 2012). The study was approved by the Ethics Committees of the University of Toyama and the Kanazawa Medical University, and the Institutional Review Board of the University of Utah.

Corresponding to the three objectives stated above, we carried out the analyses and summarized the results in three tables. In the first analysis (Table 1), we reported the distribution of KD allele and genotype frequency for the control and the KD (case) cohort. The percentage was the conditional probability of having the specific allele or genotype category. These conditional probabilities were compared between the control and case cohort, stratified by race (white, Japanese), by using the chi-square test, or the Fisher's Exact test when the frequency count was less than 5 in at least one cell in the contingency table. In the second analysis (Table 2), the association between CAL incidence and genotype was assessed using either chi-square test or Fisher's exact test. We performed four different contingency table analyses for the overall KD cohort (genotype, allele, dominant, recessive) and thus have used an adjusted type-I error by the Bonferroni method (by dividing the level of significance 0.05 by 4 which yield 0.0125). Thus, the *P*-value was considered significant if it was less than 0.0125 instead of 0.05. Similarly, we performed this analysis for the stratified cohort of Asian, and white patients. In the third analysis (Table 3), we first assessed the shape of the distribution of the continuous variable of age, CRP, WBC, and fever duration and learned using the normality test of Shapiro–Wilk and examined the histograms that these variables did not follow near normal distribution. Thus, we also reported the median in addition to the mean and standard deviation, overall, and for each of the three genotype categories. We used the nonparametric Wilcoxon rank-sum test to compare among the three groups of genotype. For gender, and treatment response to IVIG, we used either chi-square or Fisher's exact test. For this table, since all the comparisons were preplanned, and no pairwise comparisons were done, we maintained the type-I error at 0.05. All of our analyses were carried out using the SAS/STAT software version 9.3 (Cary, NC) (procedure FREQ for chi-square or Fisher's exact test, and procedure NPAR1WAY for the nonparametric Wilcoxon rank-sum test). Allelic and genotype frequencies were assessed for Hardy–Weinberg equilibrium using the online calculator at http://www.oege.org/software/hwe-mr-calc.shtml (Rodriguez et al. 2009).

**Table 1:** Allele and genotype frequencies of the SNP rs12252 (NM_021034.2:c.42T>C) in Utah Caucasian/non-Hispanic and Japanese patients with KD, compared with 1000 genome data for Utah and Japanese controls

Genotype	Controls (1000g CEU: *n* = 170)	Utah White-non Hispanic cases (*n* = 32)	*P*-value	Controls (1000g JPT: *n* = 178)	Japanese cases (*n* = 99)	*P*-value
Allele C	16 (5%)	5 (8%)	0.352	210 (63%)	121 (65%)	0.625
Allele T	324 (95%)	59 (92%)		146 (37%)	77 (35%)	
CC	0 (0%)	0 (0%)	0.358	68 (38%)	38 (38%)	0.683
CT	16 (9%)	5 (16%)		74 (42%)	45 (46%)	
TT	154 (91%)	27 (84%)		36 (20%)	16 (16%)	

*P* values were obtained by chi-square test or Fisher's exact test.

**Table 2:** The C allele and CC genotype for rs12252 (NM_021034.2:c.42T>C) are significantly associated with the development of CAL in KD patients

			Contingency table		*P* value
*All patients*					
Genotype		CC	CT	TT	
	CAL	21 (51%)	13 (26%)	10 (20%)	0.004
	No CAL	20 (49%)	37 (74%)	39 (80%)	
Allelic frequency		C	T		
	CAL	55 (42%)	33 (22%)		0.0004
	No CAL	76 (58%)	116 (78%)		
Genetic model					
Dominant		CC + CT	TT		
	CAL	34 (37%)	10 (20%)		0.039
	No CAL	57 (63%)	39 (80%)		
Recessive		CC	CT + TT		
	CAL	21 (51%)	23 (23%)		0.001
	No CAL	20 (49%)	76 (77%)		
*Asian patients*					
Genotype		CC	CT	TT	
	CAL	20 (51%)	12 (27%)	3 (19%)	0.025
	No CAL	19 (49%)	33 (77%)	13 (81%)	
Allelic frequency		C	T		
	CAL	52 (42%)	18 (23%)		0.006
	No CAL	71 (58%)	59 (77%)		
Genetic model					
Dominant		CC + CT	TT		
	CAL	32 (38%)	3 (19%)		0.164
	No CAL	52 (62%)	13 (81%)		
Recessive		CC	CT + TT		
	CAL	20 (51%)	15 (25%)		0.009
	No CAL	19 (49%)	46 (75%)		
*Caucasian patients*					
Genotype		CC	CT	TT	
	CAL	0 (0%)	1 (20%)	7 (23%)	1.000
	No CAL	1 (100%)	4 (80%)	24 (77%)	
Allelic frequency		C	T		
	CAL	1 (14%)	15 (22%)		1.000
	No CAL	6 (86%)	52 (78%)		
Genetic model					
Dominant		CC + CT	TT		
	CAL	1 (17%)	7 (23%)		1.000
	No CAL	5 (83%)	24 (77%)		
Recessive		CC	CT + TT		
	CAL	0 (0%)	8 (22%)		1.000
	No CAL	1 (100%)	28 (78%)		

*P* values were obtained by chi-square analysis.

**Table 3:** Comparison of clinical and laboratory data in KD patients with different rs12252 (NM_021034.2:c.42T>C) genotypes

Demographic	All KD (*N* = 140)	CC (*N* = 41)	CT (*N* = 50)	TT (*N* = 49)	*P* value
*All patients*					
Age at Dx (years)	2.73 ± 2.38 (2.37)	2.63 ± 2.58 (1.80)	2.76 ± 2.16 (2.00)	2.78 ± 2.45 (2.50)	0.718^1^
Gender (M/F)	94/46	29/12	29/21	36/13	0.221^2^
Second dose of IVIG required	31/139^3^	9/40	11/50^3^	11/49	0.968^2^
Laboratory data^4^					
CRP (mg/dL)	10.23 ± 7.61 (8.40)	8.71 ± 7.11 (6.37)	9.99 ± 5.82 (8.55)	11.67 ± 9.24 (8.85)	0.289^1^
WBC/*μ*L	15,103 ± 4974 (14,510)	15,347 ± 5466 (15,570)	14,174 ± 4559 (13,400)	15,752 ± 4922 (14,555)	0.303^1^
Duration of fever (days)	9.35 ± 4.90 (8.00)	10.47 ± 5.72 (8.50)	9.60 ± 4.42 (9.00)	8.27 ± 4.52 (6.00)	0.055^1^
*Asian patients*	All KD (*N* = 100)	CC (*N* = 39)	CT (*N* = 45)	TT (*N* = 16)	*P* value
Age at Dx (years)	2.62 ± 2.10 (1.90)	2.42 ± 2.07 (1.80)	2.74 ± 2.16 (2.00)	2.72 ± 2.09 (2.70)	0.607^1^
Gender (M/F)	64/36	27/12	26/19	11/5	0.503^2^
Second dose of IVIG required	23/99	8/38	11/45^3^	4/16	0.956^2^
Laboratory data					
CRP (mg/dL)	9.21 ± 6.39 (7.80)	8.28 ± 6.63 (6.37)	9.71 ± 5.84 (8.50)	9.92 ± 7.21 (6.75)	0.303^1^
WBC/*μ*L	14,427 ± 4847 (13,900)	15,047 ± 5459 (15,000)	14,116 ± 4848 (13,500)	13,869 ± 3521 (13,130)	0.566^1^
Duration of fever (days)	10.02 ± 5.22 (9.00)	10.81 ± 5.72 (9.00)	9.69 ± 4.40 (9.00)	9.20 ± 6.05 (6.00)	0.323^1^
*Caucasian patients*	All KD (*N* = 37)	CC (*N* = 1)	CT (*N* = 5)	TT (*N* = 31)	*P* value
Age at Dx (years)	2.75 ± 2.63 (2.00)	1.1 ± NA (1.1)	2.9 ± 2.44 (2.00)	2.78 ± 2.72 (2.40)	0.853^1^
Gender (M/F)	27/10	1/0	3/2	23/8	0.711^2^
Second dose of IVIG required	7/37	1/0	0/5	6/31	0.455^2^
Laboratory data					
CRP (mg/dL)	12.56 ± 9.74 (12.20)	5.10 ± NA (5.10)	11.86 ± 5.92 (13.50)	13.03 ± 10.56 (12.20)	0.747^1^
WBC/*μ*L	16,443 ± 5010 (15,250)	17,800 ± NA (17,800)	14,560 ± 2152 (13,300)	16,779 ± 5466 (15,250)	0.530^1^
Duration of fever (days)	7.76 ± 3.66 (6.00)	5.00 ± NA (5.00)	9.00 ± 5.05 (6.00)	7.64 ± 3.47 (6.00)	0.43^1^

Mean ± SD and median values (in parentheses) are reported. Dx, diagnosis; SD, standard deviation; M, male; F, female; IVIG, intravenous γ globulin; CRP, C-reactive protein; WBC, white blood cells; *μ*L, microliter; NA, not applicable (one patient).

1 *P* values obtained by nonparametric Wilcoxon rank-sum test.

2 *P* values obtained by chi-square test and Fisher's exact test when cell counts <5.

3 Data for one patient incomplete.

4 Laboratory data incomplete for 31 of the 140 patients; 9 CC, 12 CT, and 10 TT.

All 99 patients recruited in Toyama and Kanazawa were of Japanese descent. Of the 41 patients recruited in Utah, 37 were Caucasian (five with Hispanic ethnicity), 1 Asian, 1 Pacific Islander, and 2 Alaskan Native/Native American. Comparing the allelic frequencies and genotype distribution for rs12252 in the KD Caucasian/non-Hispanic and Japanese patients with 1000 genome (1000g) data from 170 Caucasian/non-Hispanic and 178 Japanese patients who did not have KD (control), did not reveal a significant difference in either (Table 1), all genotypes were in Hardy–Weinberg equilibrium. Three patients from Utah were homozygous CC, the Asian and Pacific Islander patients as well as one of the Caucasian/Hispanic patients.

Further analysis of the allelic frequencies and genotype distribution for rs12252 identified a significant association with outcome. Patients who developed CAL were significantly more likely to carry the C allele (*P* = 0.0004) and the distribution of genotypes was significantly different (*P* = 0.004) (Table 2). In addition, significantly more patients homozygous for the SNP developed CAL than patients with the other genotypes (51.2% vs. 23.2%: *P* = 0.001), supporting a recessive model for the effect of this SNP (Table 2). There was not a significant association with a dominant model (*P* = 0.039). These associations were also true when comparing outcomes in Asian patients (Table 2). There was not a significant association for Caucasians, possibly because the minor allele is very rare in this population limiting the power of the comparison in this small cohort. There were no significant differences in other clinical and laboratory data between genotypes (Table 3), including the duration of fever and the response to IVIG.

The IFITM proteins restrict the cellular entry of various viruses, including influenza A, flaviviruses, dengue virus, West Nile virus, and severe acute respiratory syndrome coronavirus (Brass et al. 2009; Huang et al. 2011). These viruses share common characteristics in that they are enveloped and enter cells via membrane fusion in endosomal compartments. It has been shown that IFITM3 prevents emergence of viral genomes from the endosomal pathway, although this may be restricted to late endosomes or lysosomes (Feeley et al. 2011). Since many enveloped viruses enter host cells through the late endocytic pathway, it is possible that enveloped viruses are an important etiologic agent in KD, particularly in patients that develop CAL. The symptoms of KD suggest that tissue damage may also occur from an over-reaction of the immune response characterized by the elevated expression of inflammatory cytokines (Saji and Kemmotsu 2006). The IFITM proteins of man and mouse have also been shown to be associated with membrane signaling complexes (Smith et al. 2006), consequently the loss of functional IFITM3 in KD patients may predispose to enhanced inflammatory responses and tissue damage.

Among the Japanese cohort, 19 (50%) of 38 patients carrying the CC genotype developed CAL. In the Utah cohort, 2 (66.7%) of 3 patients homozygous for rs12252-C developed CAL. At least in the Asian population, where the frequency of the C allele is high, screening for this SNP may be a relatively cost effective way to identify patients at higher risk of developing CAL.

In conclusion, our data reveal a novel association between the *IFITM3* rs12252 CC genotype and the development of CAL in patients with KD, particularly in Asian patients. This association did not extend to the susceptibility to develop KD but it is noteworthy that the frequency of this allele is much higher in the Asian population, as is the frequency of KD. Since this variant leads to production of a truncated protein with reduced ability to block viral release from the endocytic pathway, these data suggest enveloped viruses may be an important etiologic agent for KD and/or the development of CAL.
